# How a Philosophical Theory of Causation May Help in Ontological Engineering

**DOI:** 10.1002/cfg.258

**Published:** 2003-02

**Authors:** Daniel von Wachter

**Affiliations:** Institute for Formal Ontology and Medical Information Science University of Leipzig Härtelstrasse 16 Leipzig D-04107 Germany


**Comparative and Functional Genomics**
*Comp Funct Genom* 2003; **4**: 111–114.
Published online in Wiley InterScience (www.interscience.wiley.com). **DOI:** 10.1002/cfg.258

**Conference Review**

### How a philosophical theory of causation may help in ontological engineering

Daniel von Wachter*
*Institute for Formal Ontology and Medical Information Science, University of Leipzig, Härtelstrasse 16, D-04107 Leipzig, Germany*

*Correspondence to: Daniel von Wachter, Institute for Formal Ontology and Medical Information Science, University of Leipzig, Härtelstrasse 16, D-04107 Leipzig, Germany. E-mail: dvw@epost.de*

Received: 22 October 2002
Accepted: 5 December 2002

### Introduction

The Institute for Formal Ontology and Medical Information Science, IFOMIS (**http://ifomis.de**) at the University of Leipzig, is developing a common reference ontology for the medical domain. This ontology will be developed in tandem with work on ontology formalization being carried out at the Universities of Leeds (**http://www.comp.leeds.ac.uk/brandon/ontology/**) and Trento and Rome (**http://ontology.ip.rm.cnr.it/**) and elsewhere. It will also provide part of the ontological infrastructure for the systems for the extraction of digitalized information content from unstructured medical text that are being developed in Belgium by the company Language and Communication NV (**http://www.landc.be**).

Whilst the technology for running databases has reached an impressive state of maturity, the classification systems upon which this technology is based are the products of myriad *ad hoc* decisions stretching back to the early days of database design. The resulting inadequacies become apparent, for example, wherever attempts are made to integrate data from different sources. The problems remind one of the old fable of the Tower of Babel.

A central classification system designed to facilitate database integration was called by information scientists an 'ontology'. At IFOMIS we assume that the 2000 year-old philosophical discipline that is traditionally called 'ontology' is vital for the kind of ontology information scientists are developing. Since Aristotle's day, philosophers have been working on many of the problems that underlie the problems ontological engineers are working on. Examples of such problems concern universals and particulars, properties and relations, events and processes. At IFOMIS we assume that for building ontologies we need true philosophical theories about reality, rather than just representations of concepts or beliefs. My purpose here is to illustrate this claim by explaining how a philosophical theory of causation may help in ontological engineering.

### Philosophical theories of causation

In philosophy, two questions about causation are discussed (although they are not always distinguished). The first is what we may call the *semantic* question: can statements of the form 'A caused B' be transformed into statements of some other type? In particular, can they be transformed into statements that do not contain any reference to a cause or causes. The second is what we may call the *ontological* question: what is there in reality that makes

Copyright © 2003 John Wiley & Sons, Ltd.

true a claim that something causes something else? What in reality do we refer to in causal claims? I shall now briefly sketch some standard philosophical theories of causation and relate them to these questions. Then I shall describe the theory I favour and indicate how that theory may be relevant to the task of the ontological engineer.

Most contemporary philosophical work on causation still pays homage to David Hume's ideas. Hume considers one billiard ball pushing against another and says that we can observe the movement of the first ball and the subsequent movement of the second ball, but we cannot observe any pushing necessitating, or bringing about — in short, any *causal connexion* — between the two movements. Since he thought that every concept must be a copy of a sense impression or a complex of such copies, Hume concluded that the concept of causation does not contain the idea of a causal connexion [6]. Contemporary Humean philosophers draw the further conclusion that there is no such thing as a causal connexion. One theory of causation in this spirit is the *regularity theory*, which defines causation as follows:

> A caused B if and only if (1) A preceded B, and (2) events like A are always followed by events like B.

The resulting picture is that what happens at one time does not really have an impact on what happens later. Nothing really brings anything else about. When we call something a cause we just represent it in relation to what happens in other similar situations; we subsume it under obtaining patterns or regularities of the form: such-and-such events are always followed by such-and-such events.

This view poses obvious difficulties [7], e.g. a pattern of the given type is instantiated whenever the falling of a barometer is followed by rain, but the falling of a barometer does not cause rain. Many modifications of the regularity theory have been proposed to meet such objections. Some more sophisticated versions of the regularity theory, probabilistic theories, define causation in terms of relative frequencies: A causes B if and only if the frequency of events like B occurring subsequent to events like A is higher than the frequency of events like B occurring where no event like A occurs (i.e. A causes B if and only if $P(B|A) > P(B|\text{not-A})$ [1]. Another strategy Humeans have chosen to meet the objections that the simple regularity theory faces is to refer to regularities, not just in the actual world, but 'in all possible worlds'. The *counterfactual analysis* of causation put forward by David Lewis [2] says, in this sense, that A caused B if and only if (1) A occurred and B occurred, and (2) had A not occurred, B would not have occurred.

Regularity theories may have some plausibility if they are taken as just claiming that causal statements can be replaced by certain other statements in which no reference to causal connexions is made. One may hold that 'A caused B', at least for certain purposes, can be replaced by 'A as well as B occurred, and all events like A are followed by events like B'. But if there were no causal connexions, then probably there would not be any of the regularities to which the regularity theory refers, and probably there would not be the truths of the type 'Had A not occurred, B would not have occurred', which the counterfactual analysis uses. The answer to the semantic question of causation may be Humean even though the answer to the ontological question is non-Humean.

### The Tendency Theory of Causation

The Tendency Theory of Causation assumes that there are causal connexions and gives an ontological analysis thereof. Consider forces in Newtonian physics. If a gravitational force is acting on a planet, then that planet will move in accordance with that force if and only if there are no other forces acting on the planet. A force is an example of what I call a *tendency*. A tendency is a bias in the world at a certain time to carry on in a certain way. Forces are a kind of tendency, viz. tendencies that concern the spatial position of things.

A tendency obtains at a time $t_1$ and is a tendency towards the world carrying on after $t_1$ in a way that leads to the obtaining of a certain state of affairs, $S$, at a certain later time $t_2$. The tendency is *towards* $S$. To say that the tendency was realized is to say that things carried on in accordance with the tendency and that this led to S. A tendency will be realized if and only if there is no counteracting tendency. Some features of the world at $t_1$ are relevant for the tendency and others are not, e.g. if there is a tendency towards two planets being at certain positions at $t_2$, then the colour of the planets or the density of some remote star is not relevant for this tendency. The state of affairs at $t_1$ that is relevant

Copyright © 2003 John Wiley & Sons, Ltd. 

for the obtaining of the tendency, I call the *basis* of the tendency, e.g. where the law of gravity applies, the basis of the tendency in question is referred to in the expression: $m_1 m_2 / d^2$.

We can now say what causation is:

> A caused B if and only if A was the basis of a tendency towards B and the tendency was realized.

This theory is an answer to the ontological question about causation: it describes what it is in reality that makes causal statements true.

### An example

Let me illustrate this theory with an example. A pathologist discovers that Jones's death was caused by morphia in his blood. We identify the cause more precisely in three steps. First, we have to find out what concrete thing or stuff was involved in the causing. It was not the kidney, not the muscles, but the blood. Second, not every aspect of the blood was involved in the causing. Its consistency, the concentration of red blood cells, etc., were not relevant for the causing. Rather, it was the blood's containing morphia that was relevant. Third, in order to identify the cause completely, we have to identify the time, $t_1$ (period or instant), of the operation of the cause. So the complete identification of a cause consists of the identification of the causing *thing*, the relevant *aspect* of the thing, and the *time* of the causing. What we have identified in this way we can call a 'state of affairs' (we can also use the term 'event'). This state of affairs was the basis of a certain tendency; that is, in virtue of this state of affairs the world at $t_1$ had a bias towards carrying on in a way that included the paralysis of the respiratory system at $t_2$. This tendency was realized; there were no countervailing tendencies to stand in the way of its realization. Thus, the world carried on in accordance with the tendency, so that paralysis of the respiratory system, and hence the death of the patient, occurred. In this sense, the presence of morphia in the blood caused the patient's death. Causation, on this account, always consists in a causal process: if A caused B, then A and B were stages of the same causal process. By a *process*, I mean a continuous series of states of affairs. By a *causal process*, I mean a process each stage of which is the result of a tendency whose basis was an earlier stage of the process.

### Applications in ontological engineering

Our hypothesis at IFOMIS is that metaphysical theories of causality, space, time, material substance, body, organism, environment, spatial and temporal continuity, and so forth, can provide a common domain-neutral framework for the development of a series of domain-specific theories for ontological engineering [4,5]. In our present case this means that knowledge about the nature of causal connexions can help provide a framework for the formal representation of information about causings. Let me give four illustrations of the implications of this remark:

- First, the tendency theory entails something about what causes and effects are and how they are to be described. This tells us how we should represent causes and effects. The theory says that a tendency is 'based on a state of affairs'. This means that specifying a cause requires the specification of thing, aspect and time. Both thing and aspect may be referred to by different information systems in different ways, e.g. by using a name for the spatial location of the thing or by using a name of the thing itself (e.g. 'Jones's liver'). Formal means have to be found for unifying such different types of reports.
- Second, the tendency theory helps us to understand causation by absence (as in, 'The lack of insulin caused the hyperglycaemia') and causation by defect (as in, 'The genetic defect caused the Down's syndrome'). In both cases there is a tendency whose basis is referred to indirectly. The basis of the tendency towards the hyperglycaemia was not some 'lack', but rather some positive state of affairs involving a certain concentration of insulin at a certain time and place. Similarly, the basis of the tendency towards the Down's syndrome was not caused by a thing that is a defect, but rather by a positive state of affairs, namely by the presence of three No. 21 chromosomes.
- Third, the tendency theory entails that a tendency will be realized unless it is prevented from doing so. This gives us a clue to dealing with information about something having been prevented or

Copyright © 2003 John Wiley & Sons, Ltd. 

preventable in given circumstances. Most theories of causation assume that the occurrence of a complete cause is a sufficient condition for the occurrence of its effect. Then, however, the description of a cause becomes very lengthy, since it must refer to all things that could have prevented the effect in such a way that their non-occurrence is registered as part of the cause. From the perspective of the tendency theory, in contrast, no event is sufficient for the occurrence of a certain later event. For the theory distinguishes between a cause and possible ways in which, even in the presence of the complete cause, the effect could have been prevented. This allows more adequate and more efficient representation of causal information.

- Finally, the tendency theory may give us clues about reasoning with regard to causal information. Statistical information is to be used as evidence for there being certain tendencies in certain situations. In this way, we can acquire knowledge of the kind, 'If a patient is in state S then there is a tendency towards him having disease D (e.g. lung cancer)'. This means that, given he is in this state, he will get disease D unless something prevents that. The conjunction of 'Patient P is in state S' and 'Patient P did not develop D' entails that something prevented D. Further knowledge can be acquired about what prevents it being the case that a patient in state S develops D. Regularity theories do not allow this kind of reasoning because they do not distinguish statistical knowledge from knowledge about tendencies.

The main reason for being optimistic that, with the tendency theory, we will be able to make progress with regard to representing and reasoning with causal information is that it seems to overcome the difficulties that the regularity theories face. The objections raised in the literature suggest that regularity theories lead to false conclusions about what caused what, and they operate with generalizations that are unreliable. If we process information on the basis of an inadequate philosophical theory of causation, we should not be surprised if we get in trouble. With the tendency theory we should be more successful.

Since issues of fusion of terminologies, prevention, causation by absence and reasoning with regard to causal information are precisely among the most serious challenges facing current medical ontology [3], the tendency theory promises to be of interest not only to philosophers but also to those involved in the practical business of medical informatics.

### Acknowledgements

This work has been funded by the Alexander von Humboldt Foundation under the auspices of its Wolfgang Paul program. I thank Barry Smith and Katherine Munn for helpful comments.

### References

1. Hitchcock CR. 1997. Probabilistic causation. In *The Stanford Encyclopedia of Philosophy (Fall 2001 Edition).* Available from **http://www.illc.uva.nl/~seop/archives/fall2001/entries/causation-probabilistic/**
2. Lewis DK. 1973/1986. Causation. In *Philosophical Papers II.* Oxford University Press: Oxford, 1986; 159–213.
3. Rector AL, Rogers JE. 2000. Ontological issues in using a description logic to represent medical concepts: experience from GALEN. IMIA WG6.
4. Smith B, Varzi A. 1999. The niche. *Noûs* **33**: 198–222.
5. Smith B, Varzi A. 2002. Surrounding space: the ontology of organism–environment relations. *Theory Biosci* **121**: 139–162.
6. Strawson G. 1989. *The Secret Connexion: Causation, Realism, and David Hume*. Clarendon: Oxford.
7. Swinburne R. 1997. The irreducibility of causation. *Dialectica* **51**: 79–92.

Copyright © 2003 John Wiley & Sons, Ltd.

